# Perforated acalculous cholecystitis in a neutropenic patient following chemotherapy: a fatal case report

**DOI:** 10.1093/jscr/rjag424

**Published:** 2026-06-03

**Authors:** Yunpeng (Jack) Deng

**Affiliations:** General Surgery Department, Eastern Health, 8 Arnold St, Box Hill, Victoria 3128, Australia

**Keywords:** acalculous cholecystitis, chemotherapy-induced neutropenia, carboplatin, vinorelbine, gallbladder perforation

## Abstract

Acalculous cholecystitis is an uncommon but serious complication of myelosuppressive chemotherapy that is most often observed during profound neutropenia and carries a substantially high morbidity and mortality. This case describes the fulminant course of acalculous cholecystitis in a 78-year-old female receiving carboplatin and vinorelbine for non-small cell lung cancer, with rapid progression from initial presentation to septic shock and multiorgan failure. The case highlights the aggressive nature of acalculous cholecystitis in immunocompromised patients and underscores the importance of early clinical suspicion and prompt intervention in preventing progression to catastrophic outcomes.

## Introduction

Acalculous cholecystitis (AC) is a rare but serious inflammatory condition of the gallbladder occurring in the absence of gallstones [1]. It is most commonly seen in critically ill or immunocompromised patients [1]. Early clinical signs can be subtle and non-specific, such as abdominal pain, fever, rigours, nausea, and vomiting. These symptoms can overlap with other neutropenic abdominal emergencies such as enterocolitis and appendicitis, thereby contributing to delays in diagnosis and treatment [2, 3]. The pathophysiology of AC is multifactorial and remains incompletely understood [2].

## Case report

A 78-year-old female presented to the emergency department with a 1-day history of right upper quadrant pain associated with nausea and vomiting. She had a background of recently diagnosed right upper lobe non-small cell lung cancer, for which she had undergone a partial lobectomy three months prior. She had subsequently been commenced on adjuvant chemotherapy with carboplatin and vinorelbine and had completed three cycles of treatment at the time of presentation. An abdominal ultrasound performed the previous year had excluded gallstones. Her medical history included hypertension, vestibular neuritis, and depression. Her regular medications included esomeprazole and fluoxetine. She lived independently at home and had never smoked.

On examination, she appeared unwell and was tachycardic with a heart rate of 109 beats per minute. Abdominal examination revealed generalized tenderness with peritonism in the right upper quadrant. Investigations demonstrated severe neutropenia, with a white blood cell count of 0.2 × 10^9^/L, a neutrophil count of 0.13 × 10^9^/L, and a C-reactive protein level of 10 mg/L.

**Figure 1:**
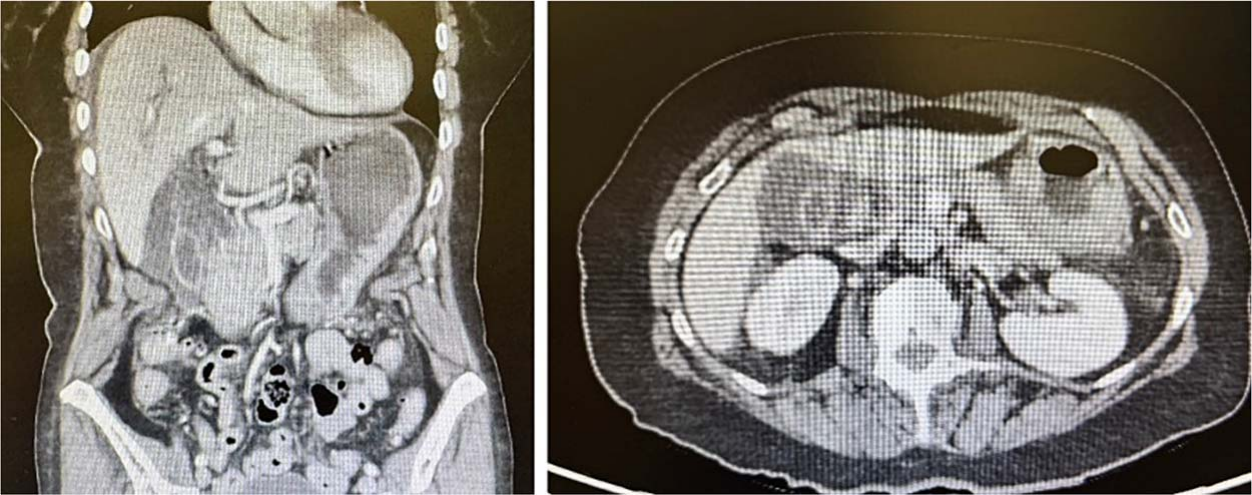
CT abdomen and pelvis with contrast. The gallbladder is severely thick walled with intramural oedema. Extensive pericholecystic soft tissue stranding is seen. The adjacent free fluid is noted extending into the subcapsular region. There are no biliary dilatation or gallstones. No concerning lesion is seen in the liver, pancreas, spleen, kidneys, or adrenal glands. No obvious hydronephrosis or renal tract calculus is seen. The bowel loops are not dilated and there is no bowel mass seen. There is no free gas or fluid seen. No abdominal or pelvic adenopathy is seen. Findings in keeping with acute cholecystitis.

Contrast-enhanced computed tomography (CT) of the abdomen and pelvis was performed and demonstrated features consistent with acute AC (Fig. 1).

The patient was resuscitated with 3 L of Hartmann’s solution and commenced on intravenous piperacillin-tazobactam. In view of persistent tachycardia and localized peritonism, a decision was made to proceed with urgent laparoscopic cholecystectomy, given concern for gallbladder perforation in an immunocompromised patient. The oncology team was consulted pre-operatively, and the patient was promptly administered a dose of filgrastim.

**Figure 2:**
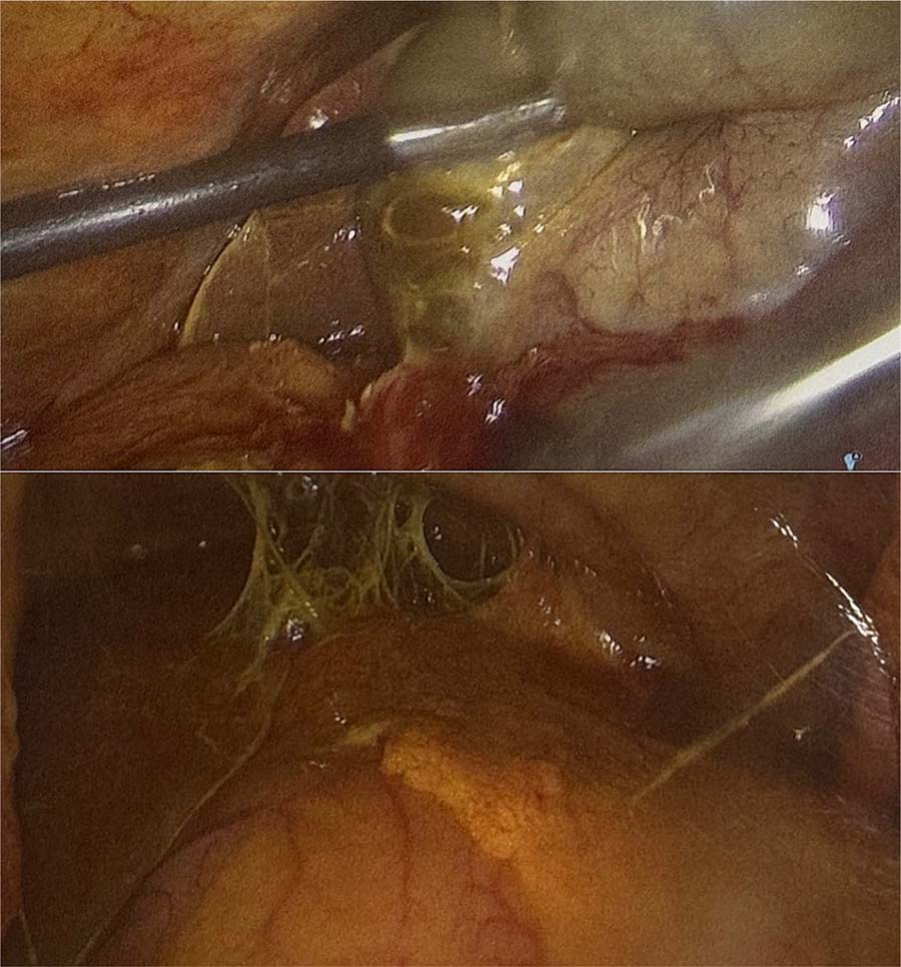
Laparoscopic view during cholecystectomy. Acute cholecystitis with perforation on the posterior gallbladder wall. Extensive bilious contamination noted in right and left upper quadrants.

The patient underwent surgery within 3 h of presentation to the emergency department. Intraoperatively, a perforated gallbladder was identified with bilious contamination of the right and left upper quadrants (Fig. 2).

**Figure 3:**
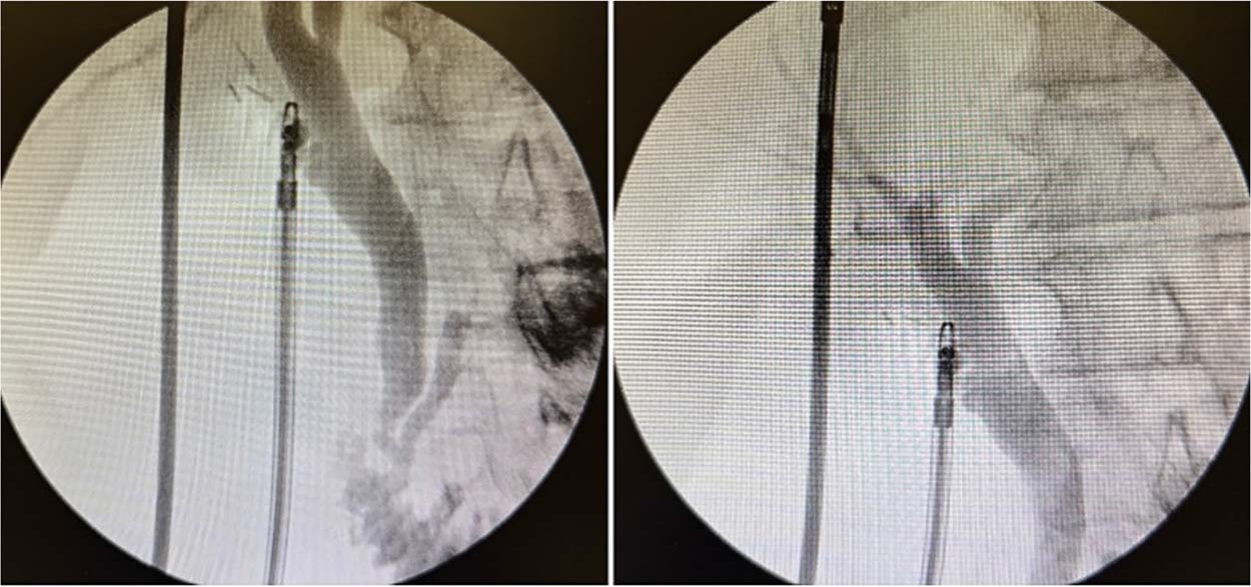
Intraoperative cholangiogram showed a dilated biliary tree with good flow into the duodenum including common bile duct, intrahepatic ducts, and pancreatic duct. There was a short cystic duct controlled with three clips.

Intraoperative cholangiography demonstrated a dilated biliary tree with unobstructed flow into the duodenum and no filling defects (Fig. 3).

The patient was transferred to the intensive care unit post-operatively. Histopathological examination confirmed acute and chronic cholecystitis with no calculi identified.

Despite early operative intervention, the patient demonstrated rapid clinical deterioration in the post-operative period. On post-operative Day 1, she remained intubated and required vasopressor support with noradrenaline and argipressin infusions. She subsequently developed atrial fibrillation with rapid ventricular response, along with acute renal and hepatic failure.

On post-operative Day 2, she developed worsening multiorgan failure and a new type 2 non-ST-elevation myocardial infarction, with a serum lactate of 6.3 mmol/L. She remained haemodynamically unstable, requiring escalating doses of noradrenaline, argipressin, and milrinone. In the context of refractory septic shock and progressive multiorgan failure, the goals of care were reviewed with the patient’s family and she was transitioned to palliative care, passing away shortly thereafter.

## Discussion

Acalculous cholecystitis is an inflammatory process of the gallbladder that occurs without gallstones and is typically associated with critical illness or immunosuppression, including sepsis, burns, trauma, major cardiovascular disorders, and haematological malignancy [1, 2]. To date, no associations between carboplatin or vinorelbine and AC have been reported in the literature [4]. The incidence of AC in the general population is ~ 2%–15% of all cases of acute cholecystitis [5]. In neutropenic patients, the reported incidence is considerably lower, ranging from 0.4% to 1.65% [6]. The mortality rate from AC can be as high as 30.4%, a figure supported by a case series by Kassar *et al.*, in which two patients developed refractory septic shock attributable to AC in the setting of chemotherapy-induced neutropenia for acute myeloid leukaemia [7, 8].

The exact pathophysiology of AC remains unclear, but proposed mechanisms include a cascade of events encompassing gallbladder stasis, ischaemia of the gallbladder wall secondary to microvascular compromise, and superimposed infection [1, 3]. In the setting of chemotherapy and its myelosuppressive effects, patients may develop profound neutropenia with associated mucosal barrier disruption, predisposing to bacteraemia or fungaemia that may seed the gallbladder. This can precipitate gallbladder ischaemia, leading to progressive wall necrosis, gangrene, and ultimately perforation [6].

Prompt diagnostic evaluation is essential in AC, and ultrasound is recommended as the first-line imaging modality [9]. Diagnostic criteria include the absence of gallstones or sludge on imaging or histopathological examination, together with features of acute cholecystitis such as gallbladder wall thickening > 3 mm, gallbladder distension, and pericholecystic free fluid [9].

Various management strategies have been described. Early laparoscopic cholecystectomy is the preferred approach in patients who are surgically fit and has been associated with superior outcomes compared to open surgery [10]. Percutaneous cholecystostomy is commonly employed in neutropenic patients who are not suitable for immediate surgery, with interval cholecystectomy performed once the patient is clinically stable [10]. Conservative management with broad-spectrum intravenous antibiotics alone is generally insufficient and is associated with risk of progression to gangrene and perforation. Therefore, early surgical intervention is pivotal in improving survival and reducing morbidity [1, 10].

A key feature of this case is the rapid clinical deterioration despite early recognition and timely surgical intervention. Within 48 h of presentation, the patient had progressed to refractory septic shock and multiorgan failure. This underscores the particularly aggressive course of AC in neutropenic patients, in whom impaired immune responses and microvascular compromise may accelerate progression to gallbladder necrosis and systemic sepsis.

## Conclusion

This case illustrates the fulminant nature of AC in the setting of chemotherapy-induced neutropenia. Despite early resuscitation and prompt surgical intervention, the patient deteriorated rapidly, developing septic shock and multiorgan failure. Clinicians should maintain a high index of suspicion for AC in neutropenic patients presenting with abdominal pain, as early recognition and timely intervention are critical to improving patient outcomes.

## Declarations

All authors concur with the contents of this submitted manuscript, and we confirm that this manuscript is not being published or considered elsewhere. The authors are either in the process of surgical training or have obtained FRACS in General Surgery.
